# A Study of Alcohol

**Published:** 1907-05-04

**Authors:** 


### A Study of Alcohol.

It is one of the pleasing and attractive features of medical thought and work that these activities can be conducted in a free atmosphere. The days of scientific orthodoxies are dead long since, and no man need now fear unpleasant consequences from the expression of his honest convictions. He may, very wisely and properly, be called on to justify these convictions, and must expect loss of reputation and influence should he fail to do so. But for sincere and earnest opinion based upon careful observation and honest reasoning, there is always an attentive audience in the medical forum. It must, however, be recognised that medical practice has, in large measure, to be conducted in circumstances which do not permit the satisfaction of all the conditions essential to scientific discussion. There is much to tempt to the expression of opinion prematurely, and before time and opportunity have been given for the judicial examination and testing of all the evidence. Indeed, there are occasions not a few in which action involving the practical acceptance of a more or less doubtful view must needs be taken. The strain of such considerations as these is probably becoming increasingly severe in medical practice. So long as tradition and empiricism governed the art of treatment, the practitioner could contentedly pursue his way according to customs which had the sanction of experience. But since the scientific observer has pushed his inquiries in such a fashion as to subject these customs to the coldly critical scrutiny of the laboratory, there must have been many anxious searchings of mind among those who undertake the treatment of disease by the administration of drugs. And when anxieties of this order have once been aroused, they can hardly be quieted until the questions at issue have been probed to their foundation.

Among drugs which have been subjected to the discipline just described, alcohol may certainly be prominently named. Enjoying in medical history, almost from time immemorial, a high reputation, its virtues have more recently been gravely questioned, and this in respect to its dietetic as well as to its medicinal use. And between the rival creeds and their respective claims, it is not to be wondered at that plain men experience considerable doubt and uncertainty. To help in the solution of this controversy we have for a number of months had an elaborate investigation conducted, and we propose to submit the details and results of this investigation to our readers. The experts who have been engaged on this work will tell their own story, and the facts they submit may be confidently accepted as the outcome of the most exact and scientific methods. The discussion of the effects of alcohol upon living tissues, and upon the functional activities of the various parts of the complex physiological mechanisms present in the most highly developed members of the animal series, ought to rest upon a basis of carefully ascertained facts. Our inquiry has been instituted and conducted for the purpose of supplying this essential element. When the facts have been ascertained, discussion about their meaning and significance may be profitable, and is to be welcomed. But hitherto there has been great freedom of assertion, both on one side and the other, while the amount of scientific observation has been limited, and even some statements claiming this distinction, have not been altogether free from the taint of partisanship.

In submitting the results of a laboratory investigation into the medicinal and dietetic value of alcohol to the judgment of the profession, we are by no means prepared to suggest that facts ascertained outside the laboratory are of little or no value. On the contrary, we regard such facts as essential to the proper settlement of the question. What is quite undoubted in certain conditions, may not apply when the conditions are changed. And a decided gain and advantage in one direction may be neutralised, or even more than neutralised, by accompanying disadvantages. What we, however, are prepared to urge is, that the alcohol controversy, like many other controversies, is made difficult and confusing because, at least to a large extent, many of the issues which it raises cannot be brought face to face with the facts out of which they are believed to grow. It therefore seems a reasonable and a proper thing, that a responsible effort should be made to clear the ground, and thus to help to a solution of an acutely contested problem. Such, at all events, is the aim of the investigation which, in due course, we shall present to our readers' judgment.

If any justification for the views here expressed were needed, it is provided by the circumstances of the present moment. The echoes of one specially engineered medical manifesto on the alcohol question have scarcely faded from our ears, when, hot upon its heels, there comes a second. Both claim distinguished professional support; yet the one is in flat contradiction to the other. The first has already served the purpose of a brewer's advertisement, and the second, without doubt, is destined to adorn many a teetotal banner. Replies and rejoinders may be expected in due course, with the inevitable result, that the question will be even more confused than it was before medical men added manifesto-making to the list of their favourite pastimes. The futility of such proceedings is obvious. Of "views" and "opinions" there are enough and to spare. What is wanted is a series of facts capable of experimental proof and demonstration. And this want we shall endeavour to supply.

